# Analysis of Preconception Paternal Smoking and Neonatal Outcomes

**DOI:** 10.1001/jamanetworkopen.2021.44527

**Published:** 2022-01-21

**Authors:** Jennifer Horwitz, Shi Wu Wen, Hongzhuan Tan, Shujin Zhou, Chang Ye, Minxue Shen, Ravi Retnakaran

**Affiliations:** 1 Leadership Sinai Centre for Diabetes, Mount Sinai Hospital, Toronto, Ontario, Canada; 2 OMNI Research Group, Department of Obstetrics and Gynecology, University of Ottawa, Ottawa, Ontario, Canada; 3 School of Public Health, Central South University, Changsha, China; 4 Liuyang Municipal Hospital of Maternal and Child Health, Beizheng, Liuyang, China; 5 Division of Endocrinology, University of Toronto, Toronto, Ontario, Canada

## Abstract

This cohort study evaluates the associations of preconception paternal smoking with neonatal outcomes.

## Introduction

There is growing emphasis on optimizing maternal health before conception to improve offspring outcomes, as per the Developmental Origins of Health and Disease paradigm.^1^ However, there has been little analogous consideration of paternal health at conception, despite growing evidence linking paternal exposures to offspring health.^2^ Indeed, the emerging Paternal Origins of Health and Disease (POHaD) paradigm posits that factors, such as paternal age and weight, can modify the sperm epigenome, yielding epigenetic changes that are maintained in the offspring, in whom they may affect gene regulation and physiology.^2^ In this context, smoking is an exposure of interest because maternal smoking in pregnancy is associated with placental DNA methylation and adverse neonatal outcomes, such as low birth weight and preterm birth.^3^ Moreover, tobacco smoke may affect the sperm genome and epigenome.^4^ However, little is known about the potential impact of preconception paternal smoking. Thus, we sought to prospectively evaluate the associations of preconception paternal smoking with neonatal outcomes.

## Methods

This cohort study was approved by the research ethics boards of Central South University in Changsha, China, Ottawa Hospital Research Institute in Ottawa, Canada, and Mount Sinai Hospital in Toronto, Canada. All participants provided written informed consent. This study has been reported in accordance with the Strengthening the Reporting of Observational Studies in Epidemiology (STROBE) reporting guideline.

In this prospective preconception observational cohort study, we recruited newly married couples in Liuyang, China, that intended to conceive within 6 months. The protocol has been described in detail.^5,6^ Between February 1, 2009, and November 4, 2015, both partners underwent baseline (pregravid) assessment and were monitored throughout pregnancy. Associations of paternal smoking with birth weight and categorical neonatal outcomes were assessed by multiple linear regression and logistic regression analyses, respectively, with *P* < .05 considered significant. We used *t* tests to evaluate the *P* values for the multiple linear regression analyses and χ^2^ tests to calculate *P* values for multiple logistic regression analyses. All tests were 2-sided, and all analyses were performed using the Statistical Analysis System version 9.4 (SAS Institute). Statistical analyses were done between January 2021 and August 2021.

## Results

The study population consisted of 1174 couples who underwent baseline assessment at median of 23.3 (IQR, 5.6-65.6) weeks before a singleton pregnancy. The mean (SD) age of the sample was 24 (3.0) years for women and 26 (3.5) years for men. At preconception assessment, 538 male partners (45.8%) reported currently smoking, in contrast to only 5 women (0.4%) doing so. The Table shows pregravid characteristics and delivery outcomes of the couples, stratified into 3 groups based on the degree of paternal smoking before conception: none (636 [54.2%]); 1 to 10 cigarettes per day inclusive (343 [29.2%]); and more than 10 cigarettes per day (195 [16.6%]). Birth weight and length of gestation did not differ between the groups.

**Table.** Characteristics of Study Population Before Pregnancy and at Delivery, Stratified Into 3 Strata of Paternal Smoking Prior to Conception

Pregravid characteristics	Participants, No. (%)			*P* value
	No smoking (n = 636)	1-10 cigarettes/d (n = 343)	>10 cigarettes/d (n = 195)	
Weeks before conception, median (IQR), wk	20.9 (4.6-63.1)	27.1 (6.6-75.8)	24.7 (7.7-67.7)	.13
Age, median (IQR), y				
Maternal	24 (22-26)	23 (22-25)	24 (22-26)	.06
Paternal	25 (24-27)	25 (23-28)	27 (24-29)	.001
Education, median (IQR), y				
Maternal	12 (9-12)	9 (9-12)	9 (9-12)	.001
Paternal	9 (9-12)	9 (9-12)	9 (9-12)	.009
Household income, 1000 yuan, median (IQR)	20 (15-30)	20 (10-30)	20 (8-30)	<.0001
Maternal smoking	4 (0.6)	0	1 (0.5)	.34
BMI, mean (SD)				
Maternal	20.2 (2.4)	20.1 (2.4)	20.3 (2.4)	.74
Paternal	22.1 (2.5)	21.8 (2.7)	22.3 (2.9)	.09
Waist circumference, mean (SD), cm				
Maternal	70.7 (7.6)	70.4 (7.7)	70.6 (7.1)	.81
Paternal	74.7 (7.6)	75.5 (8.6)	76.0 (10.5)	.11
At delivery				
Length of gestation, mean (SD), wk	39.0 (1.4)	39.0 (1.5)	39.0 (1.2)	.92
Total gestational weight gain, mean (SD), kg	17.2 (6.5)	16.9 (6.1)	17.0 (5.4)	.80
Gestational diabetes	13 (2.0)	9 (2.6)	2 (1.0)	.50
Preeclampsia	7 (1.1)	9 (2.6)	3 (1.5)	.19
Cesarean delivery	247 (39.0)	119 (35.0)	76 (39.0)	.44
1-min Apgar <7	13 (2.1)	10 (2.9)	3 (1.6)	.59
Male infant	335 (52.7)	176 (51.3)	91 (46.7)	.34
Birth weight, mean (SD), g	3295 (445)	3258 (478)	3260 (425)	.40
Adverse delivery outcomes				
Preterm delivery	23 (3.6)	21 (6.1)	6 (3.1)	.12
Low birth weight	21 (3.3)	14 (4.1)	8 (4.1)	.77
SGA	49 (7.7)	30 (8.8)	18 (9.2)	.73
LGA	78 (12.3)	37 (10.8)	17 (8.7)	.37

Abbreviations: BMI, body mass index, calculated as weight in kilograms divided by height in meters squared; LGA, large for gestational age; SGA, small for gestational age.

In the multiple linear regression analysis, positive independent factors associated with birth weight were maternal pregravid body mass index (BMI), calculated as weight in kilograms divided by height in meters squared, (regression coefficient, 39.2; 95% CI, 27.5 to 50.9; *P* < .001), paternal pregravid BMI (regression coefficient, 17.0; 95% CI, 7.7 to 26.3; *P* = .001), maternal age (regression coefficient, 14.4; 95% CI, 2.7 to 26.1; *P* = .02), length of gestation (regression coefficient, 128.7; 95% CI, 108.4 to 149.0; *P* < .001), gestational weight gain (regression coefficient, 16.5; 95% CI, 12.3 to 20.8; *P* < .0001), and male neonate (regression coefficient, 89.1; 95% CI, 36.2 to 142.1; *P* = .001), while preeclampsia was associated with lower birth weight (regression coefficient, −214.0; 95% CI, −422.9 to −5.1; *P* = .04). Neither maternal nor paternal smoking were associated with lower birth weight. Despite much higher prevalence, preconception paternal smoking was not associated with birth weight (*P* = .37). There was no significant interaction between paternal BMI and smoking status. On logistic regression analyses (Figure), neither paternal smoking exposure of 1 to 10 cigarettes per day nor smoking more than 10 cigarettes per day was associated with preterm delivery, low birth weight, small-for-gestational-age, or large-for-gestational-age.

**Figure.**
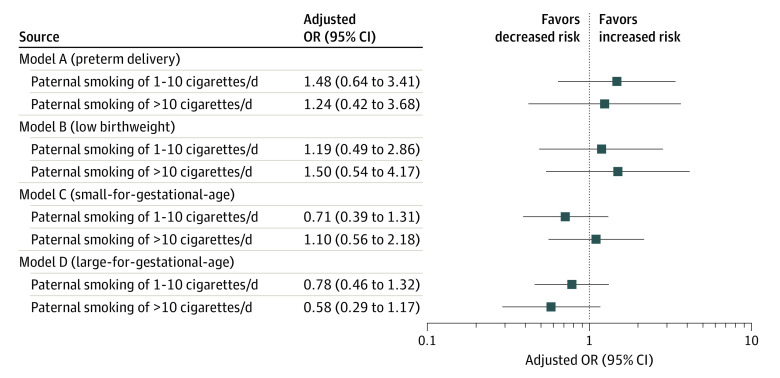
Adjusted Odds Ratios for Association of Pregravid Paternal Smoking of 1 to 10 Cigarettes Per Day and More Than 10 Cigarettes Per Day With Adverse Delivery Outcomes Each model is adjusted for pregravid paternal body mass index, calculated as weight in kilograms divided by height in meters squared, pregravid maternal body mass index, gestational weight gain, pregravid maternal smoking, and preeclampsia.

## Discussion

Previous studies have focused on paternal smoking during pregnancy as an indicator of passive maternal exposure rather than prior to conception as per the POHaD paradigm. In contrast, we have prospectively evaluated paternal smoking before conception in a population showing marked disparity in smoking rates between women and men, which enables isolated evaluation of paternal smoking. This study was limited because maternal passive smoke exposure was not assessed; however, our prospective evaluation of paternal smoking before conception does not reveal associations with birth weight or adverse neonatal outcomes at delivery. These findings cannot rule out the possibility that POHaD programming effects of preconception paternal smoking may yet emerge later in life.
